# A Superconducting Magnet UCN Trap for Precise Neutron Lifetime Measurements

**DOI:** 10.6028/jres.110.053

**Published:** 2005-08-01

**Authors:** R. Picker, I. Altarev, J. Bröcker, E. Gutsmiedl, J. Hartmann, A. Müller, S. Paul, W. Schott, U. Trinks, O. Zimmer

**Affiliations:** Technical University Munich, Physics Department E 18, D-85748 Garching, Germany

**Keywords:** beta-decay, magnetic storage, Monte Carlo simulation, neutron lifetime, superconductivity, UCN

## Abstract

Finite-element methods along with Monte Carlo simulations were used to design a magnetic storage device for ultracold neutrons (UCN) to measure their lifetime. A setup was determined which should make it possible to confine UCN with negligible losses and detect the protons emerging from β-decay with high efficiency: stacked superconducting solenoids create the magnetic storage field, an electrostatic extraction field inside the storage volume assures high proton collection efficiency. Alongside with the optimization of the magnetic and electrostatic design, the properties of the trap were investigated through extensive Monte Carlo simulation.

## 1. Introduction

The neutron lifetime *τ*_n_ plays a vital role in understanding big bang cosmology: it influences the relative abundance of primordial helium in the early universe. *τ*_n_ also opens the way to determine the coupling constants of the weak interaction and hence the element *V*_ud_ of the Cabibbo-Kobayashi-Maskawa (CKM) matrix precisely. Latest experimental results indicate this matrix to deviate from unitarity by about 3 standard deviations and therefore challenge the three-generation Standard Model [1].

The most precise measurements of *τ*_n_ were performed by storing UCN in material bottles. There are, however, significant losses during the many wall collisions whose nature is not yet fully understood. Therefore systematical errors cannot be decreased much below their present values. Magnetic storage has recently been proven to be a viable alternative [2]. This publication presents a new experiment, designed for new generation UCN sources as the one planned for the research reactor FRM II at Garching [3]. It utilizes magnetic storage in combination with real-time proton detection. Furthermore, the results of Monte Carlo calculations for the proposed setup are shown.

## 2. Description of the Experiment

Magnetic storage is based on the force ***F*** on a magnetic moment *µ* that is exerted in an inhomogeneous magnetic field with flux density ***B***

F=∇(μB) (1)

Only particles with one orientation of the spin towards ***B*** can be stored, hence reorientation (also called depolarization) has to be avoided to assure loss-less neutron storage. To reach this goal, the adiabatic condition has to be fullfilled: changes $\dot{B}$ of the magnetic field (especially rotations of the field vector) seen by the moving neutron normalized to the absolute value of the magnetic flux density *B* have to be much slower than the neutron Larmor precession in the magnetic field *ω*_L_

$$\frac{\dot{B}}{B}<<\omega_{L}.$$ (2)

**Fig. 1:**
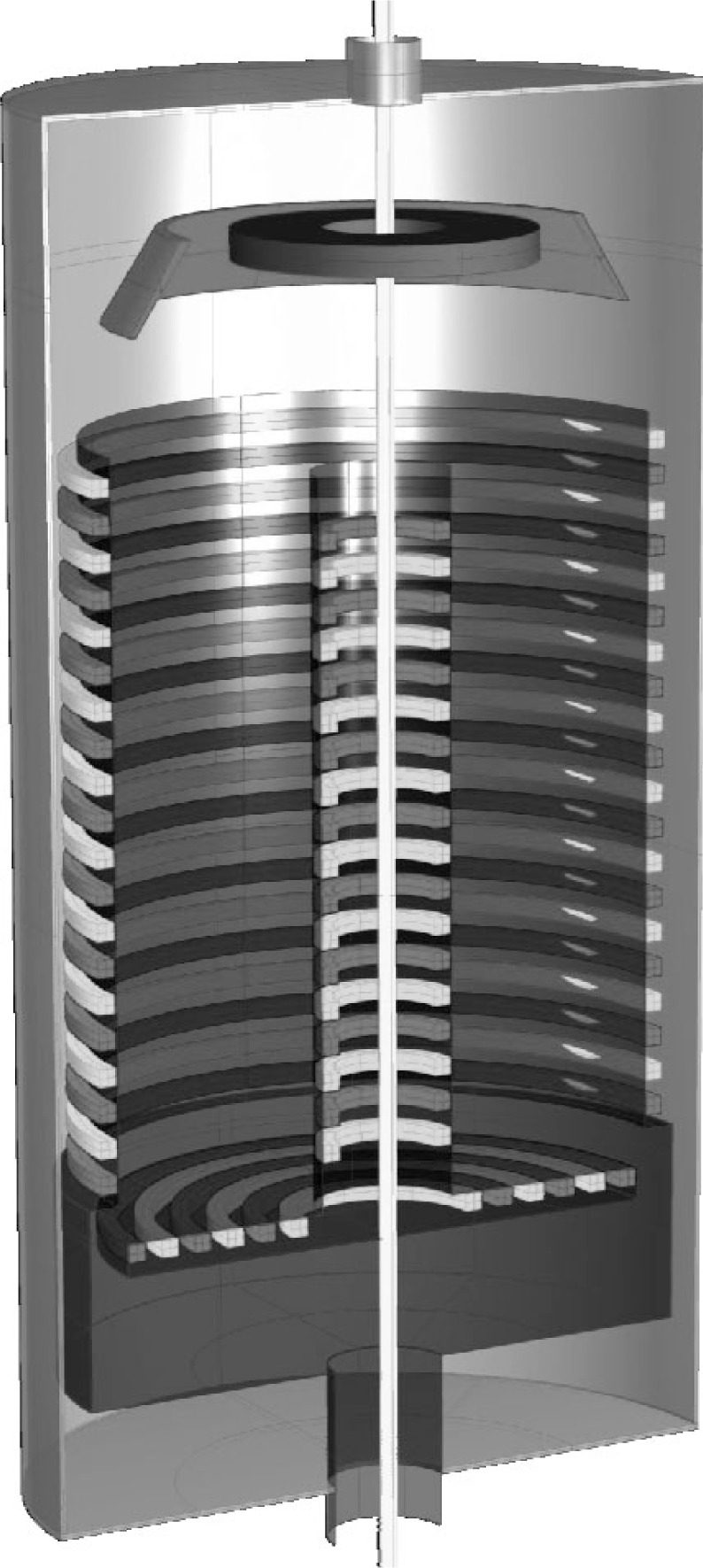
Cutaway view of the experimental arrangement. See text for further explanations.

For the proposed setup, the volume between two nested cylinders made from magnetic multipole fields is used to store the UCN (Fig. 1). This storage field is produced by stacked superconducting coils of alternating current direction with the gravitational field playing the role of the upper lid of the bottle. The dimensions of 1.2 m height and 0.5 m outer radius result in a storage volume of V ≈ 0.8 m^3^. In the center of the setup an additional current carrying rod creates an azimuthal magnetic field, which is always perpendicular to the storage field and therefore helps keep the flux density in the whole storage volume above a critical value, below which reorientation of the spin may occur [see Eq. (2)]. Filling and emptying of UCN is realized through a slit in the outer bottom corner while the current in the storage coils is low enough to let neutrons enter the trap.

*τ*_n_ shall be measured by real-time detection of decay protons as well as by counting the integral number of neutrons using different storage times. The proton detector, a CsI scintillator, is situated on top of the storage volume; the decay protons are accelerated and focused onto it through a potential difference in the storage volume and an additional focusing coil around the detector. Depolarized neutrons shall also be monitored; they are not stored magnetically, but can still be collected when the inner walls of the trap are covered with a neutron reflecting material as, e.g., diamond-like carbon. Using a new high-density UCN source at the FRM II [3] it is possible to store up to 10^8^ neutrons per cycle. Hence, the measuring time to get sufficient statistics is short and many runs with different conditions can be realized to investigate possible systematic errors. We thus envisage a relative accuracy for *τ*_n_ of 10^−4^.

## 3. Monte Carlo Simulation

**Fig. 2:**
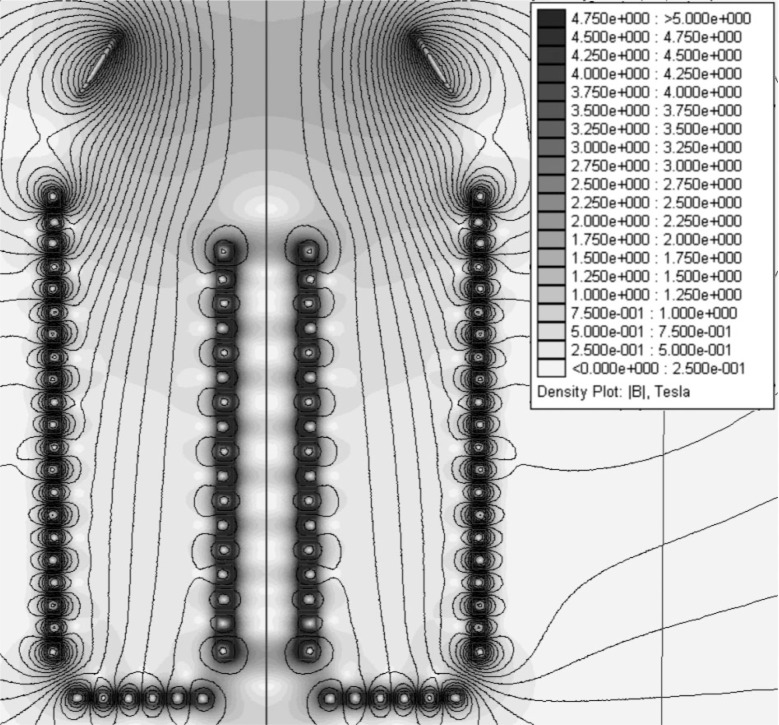
Contour plot of the magnetic flux density |***B***|, including representative lines of constant |***B***|.

**Fig. 3:**
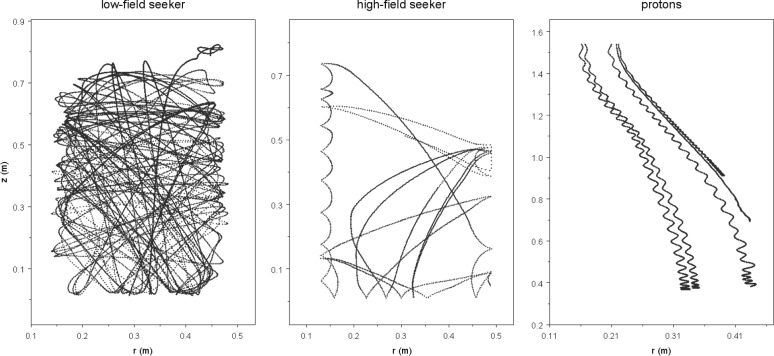
*r–z* components of example trajectories: (left) a stored neutron (*t* < 25 s)—(center) a depolarized neutron (*t* < 10 s)—(right) five decay protons.

The behavior of all particles involved in neutron decay in the experiment is dominated by the influence of the magnetic field in the case of neutrons or combined magnetic and electrostatic fields for the charged decay particles (neutrinos may be neglected). The fields (see Fig. 2) were calculated using three different finite element method programs [4–6], which produced the same results. These were then used as an input for Monte Carlo simulations. A Runge-Kutta algorithm with adaptive step-size control was used to calculate the trajectories of protons and neutrons [7]. Example trajectories for a stored neutron, a depolarized neutron and several protons are displayed in Fig. 3.

It was shown that neutrons of less than 120 neV kinetic energy can be stored loss-less inside the trap. A filling and emptying time for neutrons of less than 50 s could be confirmed as sufficient. Depolarized neutrons, arising from spin flip of the stored UCN, but also present after filling, are removed after about 25 s. When installing a neutron detector below the storage volume, they can be detected with an efficiency of around 60 %.

Another critical issue that could change the measured lifetime was also investigated: neutrons of kinetic energy *E*_kin_ higher than 120 neV have a shorter storage time, most of them (96 %) are gone after 100 s, but the ones close to 120 neV have to be cleaned by inserting an absorbing material into the storage volume at a position only accessible to UCN of *E*_kin_ > 120 neV.

Using Vladimirsky’s approach [8], the probability for UCN depolarization was found to depend on the current in the center rod: for a total current of 3 kA this probability (3 × 10^−7^) is already well below that required to reach the desired lifetime accuracy.

Protons emerging from neutron decay in the storage volume can be collected at the detector with an efficiency close to 80 %, their kinetic energies there are in the range from 30 keV to 40 keV and hence high enough for detection. The background of electrons at the scintillator was determined to be manageable, as only 2 % of all decay electrons arrive at its position. Furthermore, less than 10^−5^ of them deposit enough energy in the scintillator to be confused with the proton signal. This is again well below our accuracy goal.

## 4. Summary and Status

The neutron lifetime is a fundamental and important constant of nature. Using magnetic storage of UCN it may be measured with high precision. The feasibility of the proposed design from the physics point of view has been confirmed through Monte Carlo simulation of the involved particles. The realization is on the way, the setup is being optimized for technical feasibility and first assemblies could start later this year.
